# Production and Characterization of Fengycin by Indigenous *Bacillus subtilis* F29-3 Originating from a Potato Farm

**DOI:** 10.3390/ijms11114526

**Published:** 2010-11-12

**Authors:** Yu-Hong Wei, Li-Chuan Wang, Wei-Chuan Chen, Shan-Yu Chen

**Affiliations:** Graduate School of Biotechnology and Bioengineering, Yuan Ze University, Chung-Li, Taoyuan 320, Taiwan; E-Mails: e6501257@yahoo.com.tw (L.-C.W.); itispay@gmail.com (W.-C.C.); chensy@saturn.yzu.edu.tw (S.-Y.C.)

**Keywords:** fengycin, lipopeptide biosurfactants, media optimization

## Abstract

Fengycin, a lipopeptide biosurfactant, was produced by indigenous *Bacillus subtilis* F29-3 isolated from a potato farm. Although inhibiting the growth of filamentous fungi, the fengycin is ineffective against yeast and bacteria. In this study, fengycin was isolated from fermentation broth of *B. subtilis* F29-3 via acidic precipitation (pH 2.0 with 5 N HCl) followed by purification using ultrafiltration and nanofiltration. The purified fengycin product was characterized qualitatively by using fast atom bombardment-mass spectrometer, Fourier transform infrared spectrometer, ultraviolet-visible spectrophotometer, ^13^C-nuclear magnetic resonance spectrometer and matrix assisted laser desorption ionization-time of flight, followed by quantitative analysis using reversed-phase HPLC system. This study also attempted to increase fengycin production by *B. subtilis* F29-3 in order to optimize the fermentation medium constituents. The fermentation medium composition was optimized using response surface methodology (RSM) to increase fengycin production from *B. subtilis* F29-3. According to results of the five-level four-factor central composite design, the composition of soybean meal, NaNO_3_, MnSO_4_·4H_2_O, mannitol-mannitol, soybean meal-mannitol, soybean meal-soybean meal, NaNO_3_-NaNO_3_ and MnSO_4_·4H_2_O-MnSO_4_·4H_2_O significantly affected production. The simulation model produced a coefficient of determination (*R*^2^) of 0.9043, capable of accounting for 90.43% variability of the data. Results of the steepest ascent and central composite design indicated that 26.2 g/L of mannitol, 21.9 g/L of soybean meal, 3.1 g/L of NaNO_3_ and 0.2 g/L of MnSO_4_·4H_2_O represented the optimal medium composition, leading to the highest production of fengycin. Furthermore, the optimization strategy increased the fengycin production from 1.2 g/L to 3.5 g/L.

## 1. Introduction

As a structurally diverse group of surface-active molecules produced by microorganisms, biosurfactants have unique amphiphthic properties derived from their complex structures, including a hydrophilic moiety and a hydrophobic portion. Biosurfactants are commonly categorized as (i) glycolipids, (ii) lipopeptides, (iii) fatty acids, neutral lipids, and phospholipids, (iv) polymeric surfactants, and (v) particulate biosurfactants [1–6]. Biosurfactants have received considerable attention in recent years owing to their low toxicity, high biodegradability, enhanced environmental compatibility, high foaming ability, high selectivity as well as specific activity at extreme temperatures, pH and salinity [7]. However, biosurfactants have limited applications owing to their high production costs, which can be lowered by optimizing biosurfactant production and downstreaming processing strategies [7,8].

*B. subtilis* strains produce a broad range of bioactive peptides with a strong potential for biotechnological and pharmaceutical applications. A prominent class of such compounds is lipopeptides, including surfactin, fengycin and members of the iturin family (iturin, mycosubtilin, bacillomycin), which are amphiphilic membrane active biosurfactants and peptide antibiotics with potent antimicrobial activities [9,10]. In particular, surfactin is a thoroughly studied and well-characterized biosurfactant [11]. Such lipopeptide-type biosurfactants are characterized by their excellent surface- and membrane-active properties along with superior emulsifying and foaming properties, making them highly promising for use in food biotechnology and in the agricultural sector. Additionally, lipopeptides belonging to the iturin family are potent antifungal agents that can be used as biopesticides for plant protection [10,12].

As an antifungal, lipopeptide complex produced by *B. subtilis* strain F29-3, fengycin is a cyclic lipodecapeptide containing a β-hydroxy fatty acid with a side-chain length of 16–19 carbon atoms [12]. Particularly active against filamentous fungi, fengycin inhibits the enzymes phospholipase A2 and aromatase [12]. Similar to other lipopeptides produced by *B. subtilis*, feygycin appears as a mixture of isoforms that vary in both the length and branching of the β-hydroxy fatty acid moiety, as well as in the amino-acid composition of the peptide ring [13]. For instance, position 6 d-alanine (denoted as fengycin A) can be replaced by d-valine (denoted as fengycin B) [4,12]. Fengycin comprises two main components that differ by one amino acid exchange. Fengycin A consists of 1 d-Ala, 1 l-Ile, 1 l-Pro, 1 d-allo-Thr, 3 l-Glx, 1 d-Tyr, 1 l-Tyr, 1 d-Orn, whereas in fengyicn B, d-Ala is replaced by d-Val. The lipid moiety of both analogs is variable, as fatty acids have been identified as *anteiso-*pentadecanoic acid (*ai-*C15), *iso*-hexadecanoic acid (*i*-C16), *n*-hexadecanoic acid (*n*-C16); evidence suggests further saturated and unsaturated residues up to C18 [12,13].

This study attempts to purify fengycin produced by *B. subtilis* F29-3 through a combination of ultrafiltration and nanofiltration methods. The chemical structure of the purified fengycin is also characterized based on fast atom bombardment-mass (FAB-MS) spectrometer, Fourier transform infrared (FT-IR) spectrometer, ultraviolet-visible (UV-VIS) spectrophotometer, ^13^C-nuclear magnetic resonance (^13^C-NMR) spectrometer and matrix assisted laser desorption ionization-time of flight (MALDI-TOF). Additionally, the concentration of fengycin is assayed by performing reverse-phase HPLC analysis. Moreover, the fractions collected from the reverse-phase HPLC system are characterized based on MALDI-TOF mass spectrometry. This study also attempts to maximize the fengycin production by *B. subtilis* F29-3 in shaker flask fermentation by using statistical experimental design approaches. In addition to producing the lowest number of experimental runs, the response surface methodology (RSM) can also help to identify the effect of individual variables on medium components, evaluate the relative significance, seek the optimum constituents, and determine the factor settings that optimize the desired response, *i.e.*, fengycin production.

## 2. Results and Discussion

### 2.1. Characterization of Fengycin

#### 2.1.1. IR Spectrometric Analyses

The IR spectrum of fengycin in KBr reveals bands appearing at 3400 cm^−1^ for amino- and hydroxyl groups of amino acids. The bands appearing at 2860 cm^−1^ and 2930 cm^−1^ reflect the aliphatic side chains and at 2060 cm^−1^, the phenolic ring of tyrosine. At 1650 and 1520 cm^−1^ strong bands appeared due to the peptide bonds. The shoulder peak appearing at 1760 cm^−1^ could be attributed to an ester linkage (Figure S1(a)). The IR spectrum of fengycin from *B. subtilis* F29-3 was also consistent with the literature (Figure S1(b)) [5].

#### 2.1.2. UV Spectrometric Analyses

UV absorption maxima of the fengycin complex at 278 nm in methanol and at 293 nm in alkaline methanolic solution are indicative of tyrosyl peptides (data not shown).

#### 2.1.3. NMR Spectrometric Analyses

The ^13^C NMR spectrum exhibits carbonyl resonances between 173 and 177 ppm, both of which are carbon signals of various amino acids known from amino acid analyses. The resonances of the various fatty acid chains are found mainly between 10 and 40 ppm (Figures S2(a) and S2(c)), most of which could be assigned by a comparison with published data (Figures S2(b) and S2(d)) [14]. Some of the unsaturated carbon atoms showing resonances at 122.4 and 131.5 can be attributed to olefinic fatty acid residues.

#### 2.1.4. MALDI-TOF/MASS Analyses

For various homologues of fengycin, the signals responsible for fengycin in MALDI-TOF/MASS spectra ranged from 1435–1529 *m/z* (Table 1). During HPLC analysis, samples were collected from two to 16 minutes of elution time at one minute intervals and the collected fractions were then subjected to MALDI-TOF/MASS analysis. Table 1 summarizes the mass number of fengycin lipopeptide families observed in the MALDI-TOF mass spectra (data not shown). The mass peak appearing at *m/z* 1475.8 could be attributed to a fengycin isoform containing a β-hydroxy fatty acid with a chain length of 17 carbon atoms containing one double bond. The compounds with mass numbers of *m/z* 1497.8 and *m/z* 1505.8 were identified as fengycins with β-hydroxy fatty acid components possessing the chain lengths of 17 carbon atoms. The first species (*m/z* 1497.8) is sodium adduct of a C17 isoform with an alanine at position 6. The other compound (*m/z* 1505.8) is a protonated form of a C17 isoform with a valine instead of an alanine at position 6 (Table 1).

### 2.2. Optimization of Medium Constituents for Fengycin Production by RSM

#### 2.2.1. Fractional Factorial Design

Exactly how seven variables affect fengycin production by *B. subtilis* F29-3 was analyzed based on fractional factorial design. Table 2 summarizes the regression analysis results of the fractional factorial. The model had a coefficient of determination (*R*^2^) of 0.9109, suggesting that the sample variation exceeding 91.09% was attributed to the variables, while the model could not explain only 8.91% of the total variance. The F-value of 11.69 suggested that the model was significant. Moreover, four of the several variables examined, *i.e.*, mannitol, soybean meal, NaNO_3_ and MnSO_4_·4H_2_O, significantly affected fengycin production according to the ‘Prob > F’ value (Table 3) (considering ‘Prob > F’ values of less than 0.05 as significant). Thus, concentrations of mannitol, soybean meal, NaNO_3_ and MnSO_4_·4H_2_O were selected as independent variables to perform response surface analysis. According to the fractional factorial design, the preferable medium composition (g/L) consisted of the following: mannitol, 27.1; soybean meal, 20.8; NaNO_3_, 2.5; FeCl_2_·4H_2_O, 0.55; MgSO_4_·7H_2_O, 3.0; MnSO_4_·4H_2_O, 0.1; Na_2_MoO_4_, 0.055.

#### 2.2.2. Steepest Ascent Method

Although a highly effective means of screening variables, fractional factorial can neither estimate the optimum levels of the variables, nor determine the appropriate range of the selected variables for response surface method design. Therefore, the steepest ascent method was applied to increase fengycin production. The path of the steepest ascent was determined based on Table 4 to identify the proper direction of changing variables in order to increase fengycin production. According to this table, fengycin production was increased by elevating the concentrations of mannitol and soybean meal as well as by decreasing the concentrations of NaNO_3_ and MnSO_4_·4H_2_O. This table also revealed the yield plateau reached during the third step. Therefore, these variables were selected for further optimization via RSM design.

#### 2.2.3. Response Surface Methodology (RSM)

**Figure 1.**
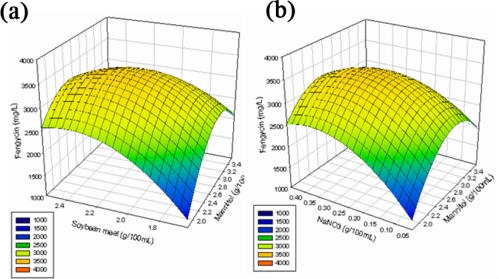

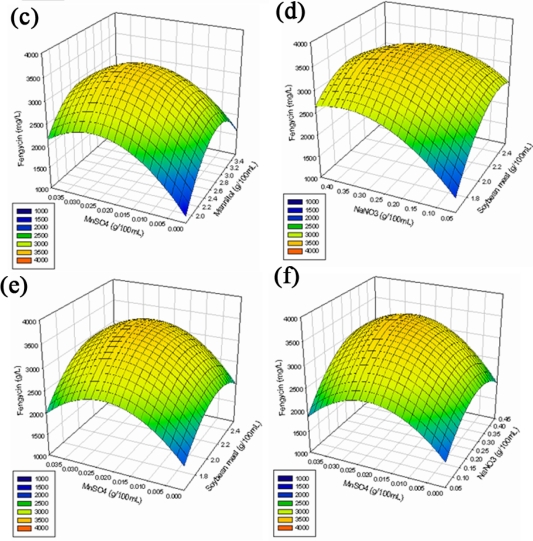
(**a**) Response surface curve based on mannitol and a soybean meal; (**b**) Response surface curve based on mannitol and NaNO_3_; (**c**) Response surface curve based on mannitol and MnSO_4_·4H_2_O; (**d**) Response surface curve based on a soybean meal and NaNO_3_; (**e**) Response surface curve based on a soybean meal and MnSO_4_·4H_2_O; (**f**) Response surface curve based on NaNO_3_ and MnSO_4_·4H_2_O.

Based on the results of fractional factorial design and the steepest ascent method, the optimal medium composition was determined based on four variables, *i.e.*, mannitol, soybean meal, NaNO_3_ and MnSO_4_·4H_2_O, which significantly influenced fengycin production, leading to optimization of fengycin production. The optimal levels of the four factors, and exactly how interactions between the four factors affect fengycin production, were determined based on central composite design (CCD) of RSM. The CCD results were analyzed by standard analysis of variance (ANOVA). Table 5 lists the mean predicted and observed responses. Thirty experiments with various combinations of mannitol (*X*_1_), soybean meal (*X*_2_), NaNO_3_ (*X*_3_) and MnSO_4_·4H_2_O (*X*_4_) were performed (Tables 5 and 6). A second order regression equation (Equation 1) describes the levels of fengycin production as a function of initial values of mannitol, soybean meal, NaNO_3_ and MnSO_4_·4H_2_O. Based on the simulation results, the response surface can be estimated by the following equation (Equation 1):

(1) $$\begin{array}{l} Y=3371.8333+18.958333X_{1}+145.125X_{2}-229.3021X_{1}^{2}-100.1875X_{2}X_{1}\\ -136.5521X_{2}^{2}-169.5417X_{3}-150.625X_{4}-139.0521X_{3}^{2}-40.3125X_{3}X_{4}\\ -194.6771X_{4}^{2}+79.0625X_{3}X_{1}+79.8125X_{3}X_{2}+48.81X_{4}X_{1}-20.6875X_{4}^{2} \end{array}$$

where *Y* refers to fengycin production, and *X*_1_, *X*_2_, *X*_3_ and *X*_4_ refers to the coded value of mannitol, soybean meal, NaNO_3_ and MnSO_4_·4H_2_O concentration, respectively. Model terms with values of ‘Prob > F’ less than 0.05 are considered significant, whereas those exceeding 0.10 are insignificant. According to the proposed model, three (*X*_2_, *X*_3_ and *X*_4_) out of the four linear terms and all of the squared model terms *X*_1_^2^, *X*_2_^2^, *X*_3_^2^, and *X*_4_^2^ were significant for fengycin production (Table 6). Coefficient of determination (*R*^2^) for fengycin production was estimated as 0.9043 (a value of *R*^2^ > 0.75 indicated the aptness accuracy of the model, which can explain up to 90.43% variability of the response. Next, the optimum level of each variable and exactly how their interactions affect fengycin production were studied by plotting three dimensional response surface curves against any two independent variables, while maintaining other variables at their respective ‘0’ levels. Figures 1(a) to 1(f) display the three dimensional curves of the estimated responses from the interaction between mannitol and soybean, mannitol and NaNO_3_, mannitol and MnSO_4_·4H_2_O, soybean meal and NaNO_3_, soybean meal and MnSO_4_·4H_2_O, and NaNO_3_ and MnSO_4_·4H_2_O, respectively. Estimated results of the response surface model equation indicated that a combination of adjusting the mannitol concentration to 26.2 g/L, increasing the soybean meal concentration to 21.9 g/L, decreasing the NaNO_3_ concentration to 3.1 g/L and adjusting the MnSO_4_·4H_2_O concentration to 0.15 g/L, would maximize fengycin production, yielding a fengycin production of 3.5 g/L. This value is significantly higher than the control value (1.45 g/L) obtained from the SMN medium, indicating that the RSM design strategy markedly improved fengycin production. Confirmation experiments based on optimal medium composition also indicated a fengycin yield of 3.55 g/L, which is consistent with the model estimates.

## 3. Experimental Section

### 3.1. Microorganism

The strain *B. subtilis* F29-3 [14–17] was a gift from Professor Shih-Tung Liu (Chang Gung University, Taiwan) and was incubated at 30 °C and 200 rpm. The cultures were stored frozen in 50% glycerol at −80 °C.

### 3.2. Growth Medium and Culture Conditions

For fengycin production, strain F29-3 was grown aerobically on a SMN medium containing (per liter) 20.0 g of soybean meal (Sigma, St. Louis, MO), 20.0 g of mannitol (Sigma), and 10.0 g of NaNO_3_ (Sigma). The pH of the medium was also adjusted with KOH (Sigma) to 7.5 ± 0.1. Erlenmeyer flask (250 mL) containing 50 mL of medium was then inoculated and incubated at 30 °C and 200 rpm for 16 h to prepare the inoculums in a rotary shaking incubator. Next, a 1% (v/v) inoculum was added aseptically to a flask (500 mL) containing 100 mL of medium. Additionally, the culture was incubated for 96 h at 30 °C and 200 rpm in a rotary shaking incubator. Finally, the growth condition was monitored by evaluating the optical density at 600 nm [14].

### 3.3. Purification of Fengycin

Following growth of strain F29-3 on SMN medium at 30 °C for 96 h, the bacterial cells were removed by centrifugation at 12000 × g for 30 min at 4 °C. The cell free fengycin was precipitated by adding 5 N HCl (Sigma) to a final pH of 2.5. The precipitates were then collected by centrifugation at 8000 × g for 10 min at 4 °C, followed by dissolution in ethanol/water (1:1, v/v, Sigma) solvent system. Next, the solution was adjusted by adding 1 N NaOH to a final pH of 7.5, and the supernatants were collected by centrifugation at 8000 × g for 10 min at 4 °C. Additionally, the filtrate was collected by filtering the supernatant through a 30 K membrane (ultrafiltration, Sigma) and, then, collecting the concentrate (about 50 mL) by filtering through a 1 K membrane (nanofiltration, Sigma). Moreover, the concentrate was diluted with 200 mL water, and fengycin was precipitated by adding 5 N HCl (Sigma) to a final pH of 2.5. Finally, the precipitate was collected by centrifugation at 12000 × g for 30 min at 4 °C and dried at 50 °C to obtain the purified fengycin powder. Our own repeat purified fengycin (*i.e.*, via acidic precipitation and followed by purification using ultrafiltration and nanofiltration more than 5 times) is used as the standard fengycin for calibration and analysis. The purity of repeat purified standard fengycin is also defined as 100%. Based on the repeat purified standard fengycin, relative purity of the purified fengycin, as applied to all the analyses, was determined as around 95%.

### 3.4. Characterization of Fengycin

#### 3.4.1. Fast Atom Bombardment-Mass Spectrometry (FAB-MS)

The purified fengycin powder was analyzed using FAB-MS (Model JMS-700, JEOL, Tokyo, Japan). FAB-MS spectra were collected over a range of 0 to 2,000 *m/z*.

#### 3.4.2. IR Spectrometric Analyses

Once identified on a KRS-5 cell, the purified fengycin powder was analyzed using Fourier transform-infrared spectrum (FT-IR) (PerkinElmer, Paragon 500, U.S.). FTIR spectra were collected between 400 and 4,000 wave numbers (per centimeter).

#### 3.4.3. UV Spectrometric Analyses

The purified fengycin powder was analyzed by UV spectrophotometry (Spectronic 601, Milton Roy, U.S.). UV spectra were collected between a range of 200 to 900 nm using methanol as the solvent and between a range of 240 to 900 nm using methanol/0.5 N NaOH (Sigma) as the solvent.

#### 3.4.4. NMR Analysis

The sample was dissolved in CD_3_OD and analyzed by NMR (Bruker, Rheinstetler, Germany).

#### 3.4.5. MALDI-TOF/MASS

The purified fengycin powder and all of the fractions collected from HPLC elutant were analyzed using MALDI-TOF/MASS (Bruker, Daltonic, Germany).

### 3.5. Quantification of Fengycin

The resulting powder was dissolved in pH of 11.0 NaOH solution (Sigma) and filtered through a PVDF syringe filters (0.45 μm) (Sigma). The culture samples were then prepared by centrifugation at 13,000 × g for 10 min to pellet the bacterial cell, followed by filtration using PVDF syringe filters (0.45 μm) (Sigma). Next, fengycin was detected and quantified by reversed-phase HPLC as follows. The above described filtrate was injected into a HPLC column (Merck), (C18, particle diameter of 5 μm, internal diameter of 4.6 m × 25 cm (length)). The mobile phase was 0.1% trifluoroaceic acid (TFA)/acetonitrile (4:6, v/v) (Sigma), and the elution rate was set at 1 mL/min. Finally, the elution absorbance was monitored at 220 nm and the injection volume was 20 μL [14,18,19].

### 3.6. Optimization of Medium Composition for Fengycin Production

#### 3.6.1. Fractional Factorial Design

The most significant parameters affecting fengycin production by *B. subtlis* F29-3 were screened based on fractional factorial design. Seven variables, *i.e.*, mannitol, soybean meal, NaNO_3_, FeCl_2_·4H_2_O, MgSO_4_·7H_2_O, MnSO_4_·4H_2_O and Na_2_MoO_4_, were studied in 16 experiments (Table 2). Each variable was represented at two levels, *i.e.*, high and low, as denoted by (+) and (−) signs, respectively. Concentration ranges for the variables were determined based on an extensive literature survey [19]. Finally, experiments were performed following the instruction of a design matrix (Table 3).

#### 3.6.2. Steepest Ascent Method

The central point and ranges of the variables that significantly influenced fengycin production used for response surface methodology (RSM) experimental design, were determined based on a single steepest ascent experiment (Table 4) [19].

#### 3.6.3. Response Surface Methodology (RSM)

Following selection of the ranges of appropriate variables, the optimum concentration of these variables was determined using RSM to increase fengycin production. Based on a central composite design (CCD) the concentration of the variables, *i.e.*, medium constitutes, was optimized along with their interactions studied. Each variable was then presented at five levels, denoted by (−2), (−1), (0), (+1), (+2), respectively (data not shown). A 2^4^ factorial design was used with eight axial points and six replicates at the center point with a total of 30 experiments (Table 5). Fengycin production was taken as the response (*Y*), and multiple regression analysis of the data was performed to derive an empirical model that relates the response measured to the independent variables. The system behavior was described according to the following quadratic equation (Equation 2):

(2) $$\begin{array}{l} Y=\beta_{0}+\beta_{1}X_{1}+\beta_{2}X_{2}+\beta_{3}X_{3}+\beta_{4}X_{4}+\beta_{11}X_{1}^{2}+\beta_{22}X_{2}^{2}+\beta_{33}X_{3}^{2}+\beta_{44}X_{4}^{2}\\ +\beta_{1}\beta_{2}X_{1}X_{2}+\beta_{1}\beta_{3}X_{1}X_{3}+\beta_{1}\beta_{4}X_{1}X_{4}+\beta_{2}\beta_{3}X_{2}X_{3}+\beta_{2}\beta_{4}X_{2}X_{4}+\beta_{3}\beta_{4}X_{3}X_{4} \end{array}$$

where, *Y* refers to the predicted response, β_0_ refers to the intercept, β_1,_ β_2,_ β_3,_ β_4_ refer to the linear coefficients, β_1,1_, β_2,2_, β_3,3_, β_4,4_ refer to the squared coefficients, β_1,2_, β_1,3_, β_1,4_, β_2,3_, β_2,4_, β_3,4_ refer to the interaction coefficients, and *X*_1_, *X*_2_, *X*_3_, *X*_4_ refer to the independent variables [20–23].

## 4. Conclusions

This study demonstrated not only that the biosurfactant produced by *B. subtilits* F29-3 is fengycin, but also that the statistical experimental designs approach markedly enhances the fengycin production. The optimal values of the tested variables to maximize fengycin production were (per liter): 26.2 g of mannitol, 21.9 g of soybean meal, 3.1 g of NaNO_3_, and 0.15 g of MnSO_4_·4H_2_O. The predicted fengycin yield was 3.5 g/L, which closely corresponds to the model estimates.

## Supplementary Material



## Figures and Tables

**Table 1.** Fengycin homologues and isoforms produced by *B. subtilis* F29-3 following growth for 96 hrs on SMN medium. The purified fengycin product was identified and quantified by reverse-phase HPLC analysis and MALDI-TOF/MASS analysis.

**Retention Time (min)**	**Main MALDI-TOF Peak(s) (*m/z*)**	**Assignment**
5, 6	1523.865, 1509.855	B-C16 and C17 fengycin [M + Na]^+^
6, 7	1509.855, 1477.828, 1491.825	B-C16 fengycin [M + Na]^+^
		A-C17 fengycin [M + H]^+^
		B-C16 fengycin [M + H]^+^
7, 8	1491.825, 1505.851	B-C16 and C17 fengycin [M + H]^+^
8, 9	1505.898, 1527.901	B-C17 fengycin [M + H]^+^
		B-C17 fengycin [M + Na]^+^
9, 10	1475.844	A-C17 fengycin [M + H]^+^
10, 11	1475.852, 1497.859	A-C17 fengycin [M + H]^+^
		A-C17 fengycin [M + Na]^+^
11, 12	1475.817, 1497.816	A-C17 fengycin [M + H]^+^
		A-C17 fengycin [M + Na]^+^
12, 13	1475.793, 1505.808	A-C17 fengycin [M + H]^+^
		B-C17 fengycin [M + H]^+^
13, 14	1511.853	B-C16 fengycin [M + Na]^+^
14, 15	1489.836	B-C16 fengycin [M + H]^+^
15, 16	1489.912	B-C16 fengycin [M + H]^+^

**Table 2.** Fractional factorial design for screening important variables that affect fengycin production (*n* = 3).

**Run No.**	**Variables**							
	**Mannitol (g/100 mL)**	**Soybean Meal (g/100 mL)**	**NaNO_3_ (g/100 mL)**	**FeCl_2_·4H_2_O (g/100 mL)**	**MgSO_4_·7H_2_O (g/100 mL)**	**MnSO_4_·4H_2_O (g/100 mL)**	**Na_2_MoO_4_ (g/100 mL)**	**Fengycin Production (mg/L)**
1	−1	−1	−1	−1	−1	−1	−1	337 ± 31
2	−1	−1	−1	1	1	1	1	1161± 104
3	−1	−1	1	−1	1	1	−1	708 ± 63
4	−1	−1	1	1	−1	−1	1	542 ± 72
5	−1	1	−1	−1	1	−1	1	447 ± 51
6	−1	1	−1	1	−1	1	−1	1688 ± 137
7	−1	1	1	−1	−1	1	1	1066 ± 101
8	−1	1	1	1	1	−1	−1	644 ± 75
9	1	−1	−1	−1	−1	1	1	1712 ± 148
10	1	−1	−1	1	1	−1	−1	1598 ± 193
11	1	−1	1	−1	1	−1	1	1054 ± 119
12	1	−1	1	1	−1	1	−1	1527 ± 124
13	1	1	−1	−1	1	1	−1	2311 ±254
14	1	1	−1	1	−1	−1	1	2527 ± 285
15	1	1	1	−1	−1	−1	−1	1556 ± 199
16	1	1	1	1	1	1	1	1853 ± 162

**Table 3.** Identifying significant variables for fengycin production using fractional factorial design a.

**Source**	**DF**	**Sum of Squares**	**F-Ratio**	**Prob > F**
Model	7	5925107.9	11.7	0.0012
Mannitol	1	3557939.1	49.1	0.0001
Soybean meal	1	745200.6	10.3	0.0125
NaNO_3_	1	500910.1	6.9	0.0302
FeCl_2_·4H_2_O	1	344862.6	4.8	0.0606
MgSO_4_·7H_2_O	1	86877.6	1.2	0.3052
MnSO_4_·4H_2_O	1	689315.1	9.5	0.0150
Na_2_MoO_4_	1	3.1	0.0	0.9950

a Coefficient of determination (*R*^2^) = 0.9109.

**Table 4.** Experimental design of steepest ascent and corresponding responses (*n* = 3).

**Experiment No.**	**Mannitol (g/100 mL)**	**Soybean Meal (g/100 mL)**	**NaNO_3_ (g/100 mL)**	**MnSO_4_·4H_2_O (g/100 mL)**
4	3.2	2.3	0.2	0.01
3	2.7	2.1	0.3	0.02
2	2.3	1.9	0.4	0.03
1	1.8	1.6	0.5	0.04
0	1.4	1.4	0.6	0.05
−1	1.0	1.2	0.7	0.06
−2	0.5	0.9	0.8	0.07

**Table 5.** Experimental design and results of central composite design (CCD) of response surface method to optimize fengycin production (*n* = 3).

**Run No.**	**Mannitol (g/100 mL)**	**Soybean Meal (g/100 mL)**	**NaNO_3_ (g/100 mL)**	**MnSO_4_·4H_2_O (g/100 mL)**	**Fengycin Production (mg/L)**	
					**Experimental**	**Predicted**
1	−1	−1	−1	−1	3033 ± 313	2956 ± 315
2	−1	−1	−1	1	2394 ± 259	2517 ± 281
3	−1	−1	1	−1	2461 ± 216	2218 ± 215
4	−1	−1	1	1	1981 ± 238	1941 ± 221
5	−1	1	−1	−1	3351 ± 315	3327 ± 381
6	−1	1	−1	1	2699 ± 289	2807 ± 252
7	−1	1	1	−1	2682 ± 278	2909 ± 264
8	−1	1	1	1	2623 ± 242	2550 ± 281
9	1	−1	−1	−1	2867 ± 236	2938 ± 312
10	1	−1	−1	1	2968 ± 256	2695 ± 261
11	1	−1	1	−1	2613 ± 281	2516 ± 274
12	1	−1	1	1	2414 ± 261	2435 ± 253
13	1	1	−1	−1	2858 ± 275	2909 ± 287
14	1	1	−1	1	2343 ± 284	2584 ± 261
15	1	1	1	−1	2933 ± 213	2807 ± 271
16	1	1	1	1	2554 ± 215	2643 ± 284
17	0	0	0	0	3263 ± 326	3371 ± 391
18	0	0	0	0	3418 ± 321	3371 ± 337
19	0	0	0	0	3297 ± 323	3371 ± 312
20	0	0	0	0	3449 ± 324	3371 ± 353
21	−2	0	0	0	2413 ± 211	2416 ± 252
22	2	0	0	0	2506 ± 230	2492 ± 240
23	0	−2	0	0	2302 ± 220	2535 ± 311
24	0	2	0	0	2242 ± 254	3115 ± 335
25	0	0	−2	0	2375 ± 257	3154 ± 291
26	0	0	2	0	2352 ± 215	2476 ± 245
27	0	0	0	−2	2782 ± 248	2894 ± 281
28	0	0	0	2	2414 ± 261	2291 ± 322
29	0	0	0	0	3379 ± 357	3371 ± 352
30	0	0	0	0	3425 ± 322	3371 ± 336

**Table 6.** Model coefficients estimated by multiple linear regression analysis a.

**Source**	**Coefficient**	**Standard Error**	**t-Value**	**Prob > t**
Intercept	3371.8	74.6	45.2	<0.0001
*X*_1_	18.958	37.3	0.5	0.6184
*X*_2_	145.1	37.3	3.9	0.0014
*X*_3_	−169.5	37.3	−4.6	0.0004
*X*_4_	−150.6	37.3	−4.0	0.0011
*X*_1_*X_1_	−229.3	34.9	−6.6	<0.0001
*X*_2_*X_1_	−100.2	45.7	−2.2	0.0444
*X*_2_*X_2_	−136.6	34.9	−3.9	0.0014
*X*_3_*X_1_	79.1	45.7	1.7	0.1038
*X*_3_*X_2_	79.8	45.7	1.8	0.1009
*X*_3_*X_3_	−139.1	34.9	−4.0	0.0012
*X*_4_*X_1_	48.8	45.7	1.1	0.3019
*X*_4_*X_2_	−20.7	45.7	−0.5	0.6569
*X*_4_*X_3_	40.3	45.7	0.9	0.3912
*X*_4_*X_4_	−194.7	34.9	−5.6	<0.0001

a Coefficient of determination (*R*^2^) = 0.9043.
