# Supplementary material for: Production and Characterization of Fengycin by Indigenous Bacillus subtilis F29-3 Originating from a Potato Farm
Source: Int J Mol Sci. 2010 Nov 12;11(11):4526–38. doi: 10.3390/ijms11114526 (PMC3000098; doi:10.3390/ijms11114526)

**Figure S1.** (**a**) Infrared spectrum for fengycin product obtained in this work; (**b**) Infrared spectrum of fengycin from literature [15].

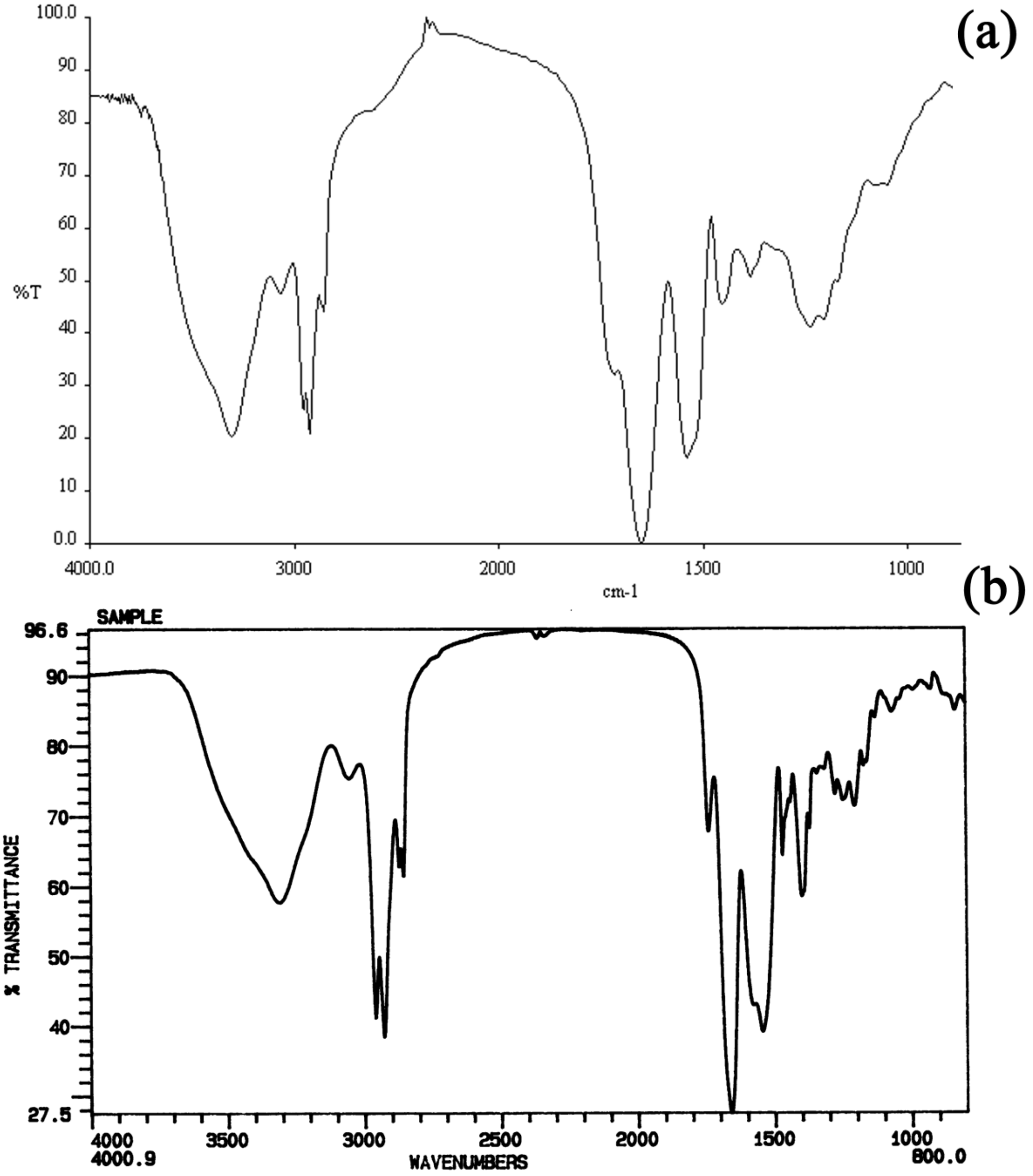

**Figure S2.** (**a**) $^{13}$C-NMR spectrum for fengycin product obtained in this work; (**b**) $^{13}$C-NMR spectrum of fengycin from literature [14]; (**c**) $^{13}$C-NMR spectrum for fengycin product obtained in this work; (**d**) $^{13}$C-NMR spectrum of fengycin from literature [14]

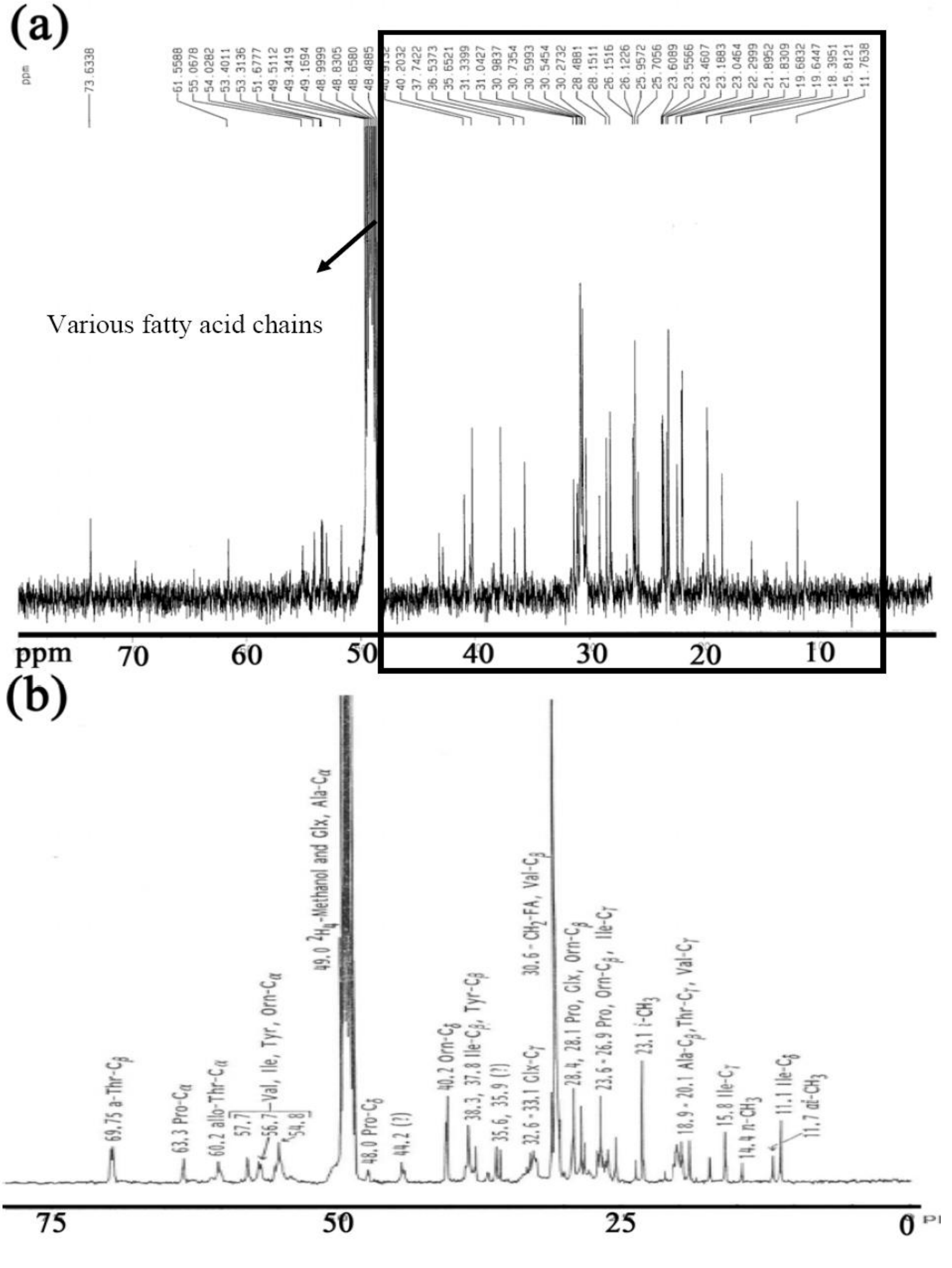

**Figure S2.** *Cont.*

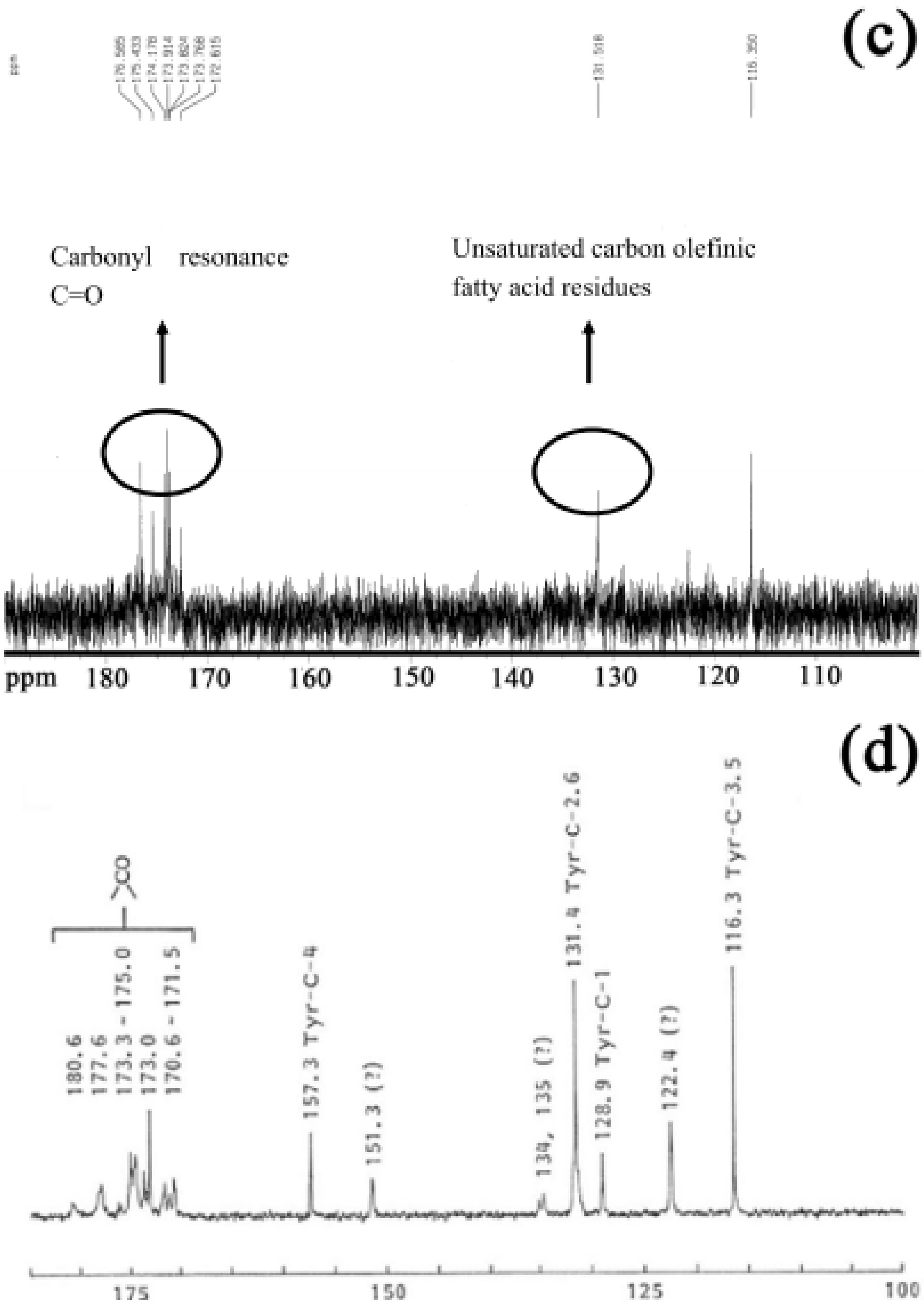

Supplement: Supplementary file 1 [file ijms-11-04526-s001.pdf]
